# The Size-Dependent Effects of Silver Nanoparticles on Germination, Early Seedling Development and Polar Metabolite Profile of Wheat (*Triticum aestivum* L.)

**DOI:** 10.3390/ijms232113255

**Published:** 2022-10-31

**Authors:** Lesław Bernard Lahuta, Joanna Szablińska-Piernik, Karolina Stałanowska, Katarzyna Głowacka, Marcin Horbowicz

**Affiliations:** Department of Plant Physiology, University of Warmia and Mazury, Genetics and Biotechnology, Oczapowskiego Street 1A/103, 10-719 Olsztyn, Poland

**Keywords:** wheat, seedling, silver nanoparticles, metabolic profiles

## Abstract

The phytotoxicity of silver nanoparticles (Ag NPs) to plant seeds germination and seedlings development depends on nanoparticles properties and concentration, as well as plant species and stress tolerance degrees. In the present study, the effect of citrate-stabilized spherical Ag NPs (20 mg/L) in sizes of 10, 20, 40, 60, and 100 nm, on wheat grain germination, early seedlings development, and polar metabolite profile in 3-day-old seedlings were analyzed. Ag NPs, regardless of their sizes, did not affect the germination of wheat grains. However, the smaller nanoparticles (10 and 20 nm in size) decreased the growth of seedling roots. Although the concentrations of total polar metabolites in roots, coleoptile, and endosperm of seedlings were not affected by Ag NPs, significant re-arrangements of carbohydrates profiles in seedlings were noted. In roots and coleoptile of 3-day-old seedlings, the concentration of sucrose increased, which was accompanied by a decrease in glucose and fructose. The concentrations of most other polar metabolites (amino acids, organic acids, and phosphate) were not affected by Ag NPs. Thus, an unknown signal is released by small-sized Ag NPs that triggers affection of sugars metabolism and/or distribution.

## 1. Introduction

The growing interest in the use of silver nanoparticles (Ag NPs) in various industries (medicine, textile, cosmetics, environment, construction, food packaging, printing, electronics, and home appliance) leads to an increase in their production and usage [1,2]. Due to their antiviral, bactericidal, and fungicidal properties, Ag NPs are used in medicine for diagnosis, treatment, and drug delivery, as well as medical device coating, wound dressings, medical textiles, bone healing, bone cement, dental applications, and coating materials for cardiovascular implants and catheters [3,4,5,6,7]. The toxicity of Ag NPs to microorganisms makes them also suitable materials for plant protection against bacteria, fungi, viruses, and insect pests in agricultural practice [8,9,10]. Moreover, Ag NPs can be used as growth stimulants for crops [11] and improvement of their abiotic stress tolerance [12]. However, the use of silver nanoparticles in crops can also be a threat to the plants themselves.

Plants respond to Ag NPs in a dose-dependent manner, promoting or inhibiting growth [13,14,15,16]. For example, Ag NPs at concentrations below 40 mg/L can stimulate germination and seedlings growth of some legume species [15,17,18,19]. In contrast, the inhibition of seedlings growth along with increasing concentrations of Ag NPs was found in *Arabidopsis thaliana* [18] and also in legumes [15,20] and cereals [21,22,23,24,25].

The mechanism of phytotoxicity of Ag NPs against plant cells appears to be similar to that caused against microorganisms [13,26,27]. Currently, it is believed that both the concentration and size of Ag NPs are the major factors affecting their phytotoxicity in plants [13,15,16,28] and in other organisms [29,30,31]. The toxic concentrations of Ag NPs on single-celled organisms are in the range of 0.1–20 mg/L, whereas they are 10–100 mg/L on eucaryotic cells in vitro [29]. Ag NPs of smaller size show higher toxicity than larger ones, presumably due to the easier penetration into cells and higher release of harmful silver ions (Ag^+^) from their surface [13,26,27,29,32,33,34]. In *A. thaliana* grown in hydroponic media, the inhibition of roots elongation by citrate-stabilized Ag NPs was much stronger by 20 nm nanoparticles than by those of 80 nm diameter, at concentrations in the range 66.84–534.72 µg/L [13]. Similarly, in common grass, Ag NPs with a diameter of 6 nm and a concentration of 40 mg/L more strongly inhibited seedling growth than those with a diameter of 25 nm [34]. Presumably, small nanoparticles more easily penetrate the pores in the cell walls of the root epidermal cells, which enables their further translocation via apoplastic and symplastic pathways into vascular bundles and then throughout the whole plant [13,35,36,37,38]. The toxicity of Ag NPs to plants also depends on other nanoparticle’s properties that are related to their methods of synthesis (chemical, physical, or biological), coating/stabilizing agents, application routes (i.e., on seeds before sowing, on leaves/shoots, or into the soil during plants vegetation), and on the physiology and multifaceted anatomy of particular plant species [14,15,16,39,40,41].

In studies on the sensitivity of germinating seeds to Ag NPs and/or Ag^+^ ions, the toxic effects of silver are noted firstly in the root tips and then in shoots; for review see [16,26,27,28,38,42]. This is probably caused by the translocation of silver ions from roots to shoots [35,36,43]. Although a common reaction of plant cells to Ag NPs applied at toxic concentrations is an oxidative burst, with consequent lipid peroxidation and membrane damage, much less is known about changes in their transcriptome, proteome, and metabolome. Some data indicate that the Ag NPs or Ag^+^ can induce transcriptional and proteomic changes [44,45], an increase in antioxidant enzyme activity, and disruption of both primary [44,46,47] and secondary metabolism [45].

Our previous study [48] showed growth inhibition of wheat (*Triticum aestivum* L.) seedlings and changes in their primary metabolism by biosynthesized silver nanoparticles ((Bio)Ag NPs) at low concentrations (20–40 mg/L). However, the size of these (Bio)Ag NPs was not uniform: 75% of the counted nanoparticles ranged between 5 and 10 nm and ca 20% between 15 and 25 nm [48]. Moreover, their surface coating agents are not identified in detail so far [49]. Thus, for a closer explanation of the relationships between Ag NPs size/coating agents and their phytotoxicity to wheat seedlings, the effects of chemically synthesized citrate-stabilized monodispersed spherical Ag NPs of 10, 20, 40, 60, and 100 nm in diameter (at a concentration of 20 mg/L) on wheat grain germination and early seedlings development and their metabolic profiles were compared. Obtained results reveal the major changes in primary metabolism in response to stress caused by Ag NPs.

## 2. Results and Discussion

### 2.1. The Effect of Ag NPs on Germination and Early Seedling Development

**Figure 1:**
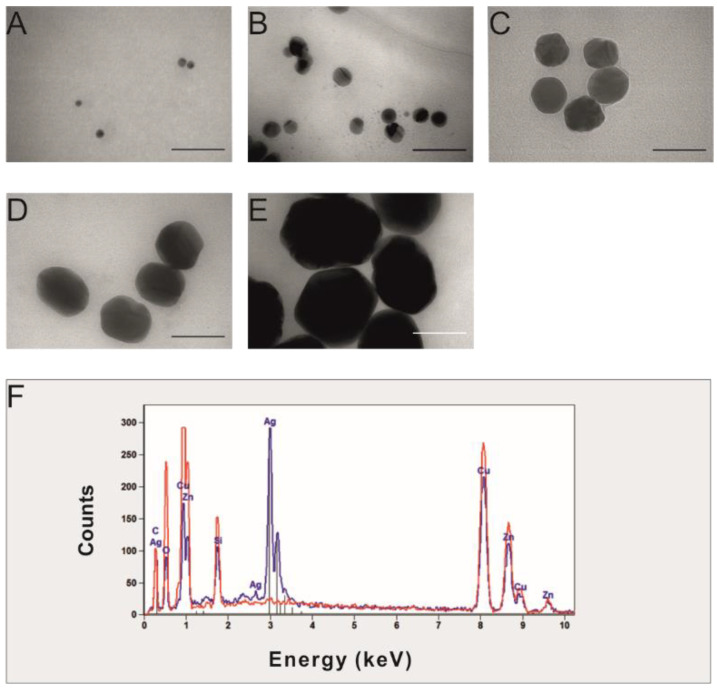
Transmission electron microscopy (TEM) image of Ag NPs (**A**–**E**) and energy dispersive X-ray spectroscopy (EDS) analysis (**F**) of Ag NPs (blue) and corresponding grid (red). Horizontal bars (on **A**–**E**) correspond with 50 nm.

The size and shape of Ag NPs used in the present study are shown in Figure 1A–E. The Ag NPs with sizes of 10, 60, and 100 nm were mostly monodispersed (10.4 ± 2.5; 63.1 ± 8.5, and 91.4 ± 9.2 nm), while those of 20 and 40 nm Ag NPs had sizes lower than declared by the manufacturer (15.4 ± 2 and 34.8 ± 4 nm, respectively, Figure S1). The smallest particles (10 and 20 nm) were mostly regular round, while the shape of the larger NPs (60 and 100 nm) was less regular as they were elliptical or angular. Energy-dispersive X-ray spectroscopy confirmed the presence of metallic silver registered at 2.7–3.2 keV (Figure 1F), as well as copper and zinc elements (from TEM grinding used for the analysis).

**Figure 2:**
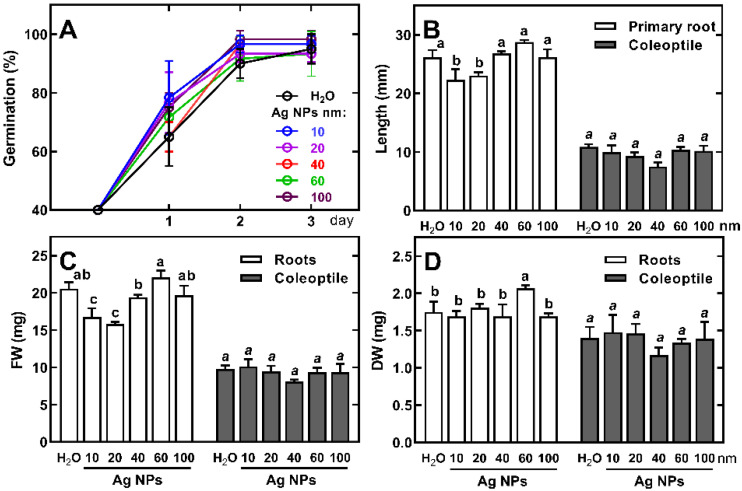
The effect of citrate-stabilized Ag NPs (at 20 mg/L) with various sizes (in the range of 10–100 nm) on the germination of the grain (**A**), length of primary seminal root and coleoptile (**B**), fresh weight (FW, **C**), and dry weight (DW, **D**) of roots and coleoptile of 3-day-old seedlings of wheat (*Triticum aestivum* L. cv. ‘Ostka Strzelecka’). Values are means (*n* = 3) ± SD (**A**) or + SD (**B**–**D**). The same letters above the bars (**C**,**D**) indicate no significant (*p* < 0.05) differences after the ANOVA and Tukey’s post hoc corrections.

Wheat grains germinated well in the presence of Ag NPs (Figure 2A), regardless of their sizes. However, the growth of the primary seminal root was inhibited by 10 nm and 20 nm Ag NPs (Figure 2B). Moreover, the fresh weight (FW) of roots also decreased (Figure 2C), although without significant changes in their dry weight (DW, Figure 2D). Ag NPs did not affect FW and DW of the coleoptile, but there was a tendency to inhibit the growth of this organ in the presence of small-sized Ag NPs (10–40 nm, Figure 2B).

**Figure 3:**
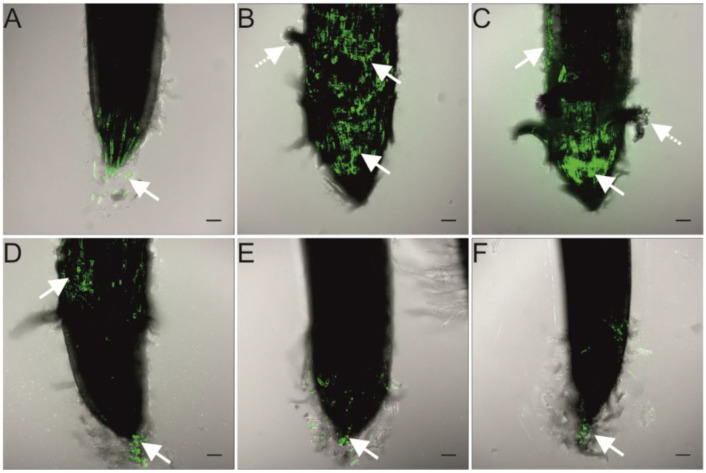
Determination of cellular reactive oxygen species (ROS) by H_2_DCF-DA assay in the wheat root tips after 3 days of seedling development in Ag NPs with sizes of 0 (**A**), 10 (**B**), 20 (**C**), 40 (**D**), 60 (**E**), and 100 nm (**F**). Scale: the length of the horizontal white bars equals 100 µm. The white arrows show the higher level of ROS generation.

The major deteriorations of cells, caused by Ag NPs, were found in root tips of wheat seedlings treated with the smaller Ag NPs (10, 20, and 40 nm). The H_2_DCF-DA, used to show the generation of the reactive oxygen species (ROS), revealed the green fluorescence (indicating ROS presence) in all analyzed root tips (Figure 3). However, the localization of ROS depended on the size of the Ag NPs used in the experiment. In roots of seedlings treated with the smallest Ag NPs (10 and 20 nm), the ROS fluorescence was noted in the apical meristem region and elongation zone (Figure 3B,C, arrows). In seedlings treated with a medium-sized Ag NPs (40 nm), the ROS fluorescence was observed both in the elongation zone and cap cells (Figure 3D, arrows), whereas it was observed only in the cap cells of non-treated seedlings and those treated with the larger Ag NPs (60 and 100 nm, Figure 3A,E,F, arrow).

**Figure 4:**
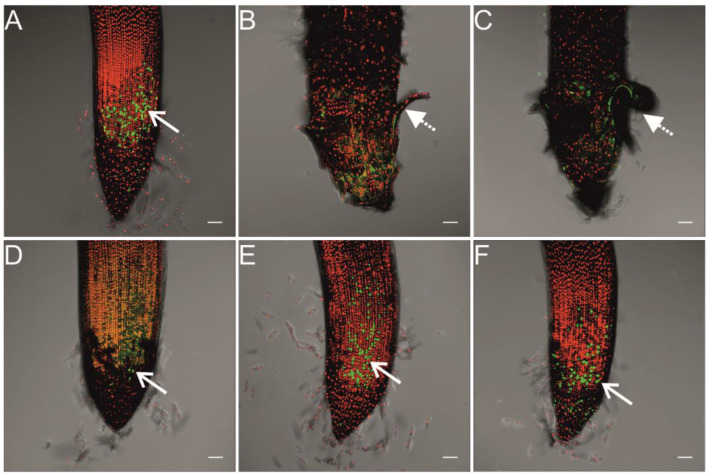
Fluorescence images of a live/dead assay of the wheat root tips after 3 days of seedling development in Ag NPs with sizes of 0 (**A**), 10 (**B**), 20 (**C**), 40 (**D**), 60 (**E**), and 100 nm (**F**). Syto9 green fluorescence is characteristic for viable cells and nonviable cells, and PI red fluorescence is characteristic for nonviable cells only. Scale: the length of the horizontal white bars equals 100 µm. In seedlings treated with 10 and 20 nm Ag NPs, more dead than live cells were localized in the root apical region (**B**,**C**), dotted arrows with a higher level of ROS generation (Figure 3B,C, dotted arrows).

The generation of ROS was compatible with the localization of dead cells (Figure 4). The live and dead cells in the root tips of the non-treated seedlings occurred in the same regions as in seedlings treated with 60 and 100 nm but also 40 nm Ag NPs (Figure 4A,D–F).

The effect of Ag NPs on the germination and development of wheat seedlings seems to be related to both concentration of Ag NPs and the coating agents. In our previous study, the mixture of small-sized (Bio)Ag NPs (5–25 nm) at the concentration of 20 mg/L decreased the early growth of wheat primary seminal root by 50% and coleoptile by 25% [48]. A similar effect was also found by Vannini et al. [46], who applied 10 nm PVP-Ag NPs at a concentration of 10 mg/L. Moreover, both chemically and green-synthesized Ag NPs (with sizes of 25–70 and 7.5–25 nm, respectively) at concentrations in the range of 10–50 mg/L had genotoxic effects on wheat root tip cells [25]. However, the citrate-stabilized 15 nm Ag NPs at a concentration of 50 mg/L were not toxic for the wheat [26]. The present study on the treatment of wheat grains with Ag NPs stabilized with citrate can show the relationship between nanoparticles size and their phytotoxicity. Indeed, a slight inhibition of roots growth by the small-sized Ag NPs (10–20 nm, Figure 2B,C), as well as the higher generation of ROS and dead cells in root tips (Figure 3 and Figure 4), confirms their phytotoxicity [35,36,37,38]. However, the present study showed weaker inhibition of wheat root and coleoptile growth by citrate-stabilized Ag NPs than by (Bio)Ag NPs [48]. This leads to the supposition that coating agents play an important role in the phytotoxicity of Ag NPs. This seems to be due to the interaction with the electrical charge of the surface of NPs which affects their adhesion in tissues. In other words, negatively charged, citrate-coated Ag NPs are easier to accumulate in Gram-positive bacteria than in Gram-negative bacteria [50]. In addition, cystamine-stabilized and positively charged Ag NPs were previously found to be more cytotoxic to wheat cells in vitro than citrate-stabilized Ag NPs [51]. In addition, it cannot be excluded that the phytotoxic effect is related to the structural properties of plant cell wall components and the corresponding rapid electrostatic interaction between the surface NPs and the plant cell wall and plasma membrane [52]. Other factors, such as cell membranes carrier proteins, Casparian strip barrier in the endodermis, the structure of plasmodesmata, and cells in vascular bundles, also are involved in the process of Ag NPs internalization [35,36,37,38,43]. Pradas del_Real et al. [53] showed preferential accumulation of Ag NPs in the discontinuity between root epidermal cells, while inside the roots (cell walls of the cortex cells, endodermis, and in the central cylinder) Ag-thiol and other ionic Ag species were detected, instead of metallic and elemental silver. It means, that Ag NPs were completely dissolved and complexed by plant organic ligands, among them various organic acids [54] and sulfur-rich proteins [55]. Moreover, Ag NPs absorbed by roots are translocated inside plants [35,36,37,38] and undergo biotransformation, including aggregation, oxidative dissolution, sulfidation, chlorination, and complexation with organic matter, such as thiolates [43,56,57]. Therefore, the physiological effects of Ag NPs can be associated with various signals triggered by Ag NPs themselves and by the products of their transformation.

### 2.2. Profile and Content of Polar Metabolites in Wheat Seedlings Organs

The same set of polar metabolites was detected in wheat seedlings as in our previous studies [48]. They were represented by both, products of degradation of reserve materials (carbohydrates, protein amino acids, and phosphoric acid) and intermediates in primary metabolism (i.e., organic acids and phosphate; Table 1).

Among soluble carbohydrates, monosaccharides (glucose, fructose, and galactose) and sucrose (in roots and coleoptile) or maltose (in endosperm) were predominant, and among amino acids, glutamine and asparagine. The major organic acids were malate and citrate.

The concentration of total identified polar metabolites (TIPMs) in control seedlings of wheat was ca twofold higher in roots (107.70 mg/g DW) and coleoptile (129.69 mg/g DW) than in endosperm (56.08 mg/g DW). The soluble carbohydrates were the major polar metabolites, sharing 42, 73, and 91% of TIPMs, in roots, coleoptile, and endosperm, respectively. Among soluble carbohydrates, fructose, and glucose (in roots and coleoptile) or maltose and sucrose (in the endosperm) were quantitatively dominant. In both, roots and coleoptile, the major amino acids were glutamine and asparagine (Table 1). The concentrations of total amino acids (TAAs) and total organic acids (TOAs, represented mainly by citrate and malate) were much higher in roots (35.61 and 17.16 mg/g DW for TAAs and TOAs, respectively) than those in coleoptile (17.16 and 9.09 mg/g DW) and endosperm (3.00 and 1.11 mg/g DW).

The composition of polar metabolites in wheat seedlings highlights the mobilization of various storage materials during grain germination and seedlings development. The degradation of starch in wheat endosperm during grain germination leads to the release of dextrins and short-chain products, such as maltotriose, maltose, and finally glucose [58,59]. Indeed, the latter three metabolites were found in the endosperm of the examined wheat cultivar (Table 1). Moreover, the presence of sucrose, as well as 1-kestose in roots and coleoptile of wheat, confirms both the rapid synthesis of sucrose and its transport [58] and activation of the biosynthesis of fructans in vegetative tissues [60], respectively. Additionally, proteolysis of storage proteins leads to an increase of free amino acids, among them glutamine and asparagine, which are among the major amino acids translocated in wheat [61] and other plants [62].

The presence of Ag NPs during wheat germination and early seedling development resulted in a slight decrease in TIPMs only in coleoptiles (except for 60 nm Ag NPs, Figure 2A), which was due to reduced levels of total soluble carbohydrates (TSCs, Figure 2B).

**Figure 5:**
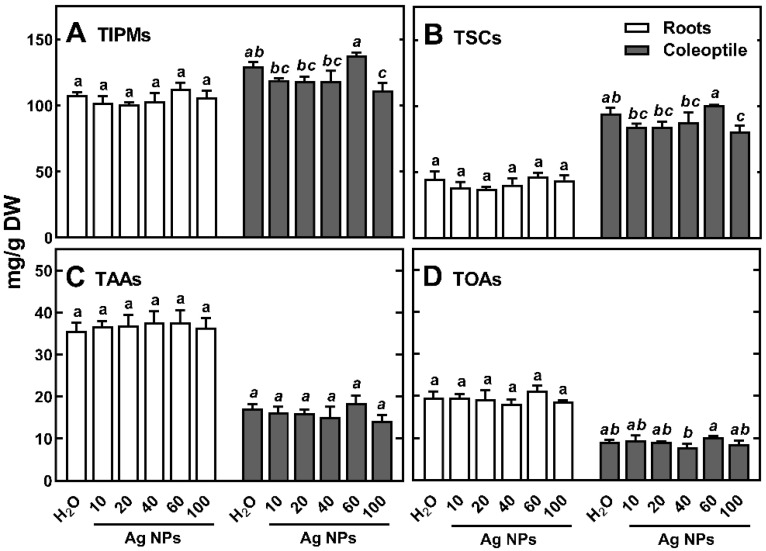
The effect of various sized Ag NPs citrate-stabilized at 20 mg/L on the concentration of total identified polar metabolites ((**A**), TIPMs), including total soluble carbohydrates ((**B**), TSCs), total amino acids ((**C**), TAAs), and total organic acids ((**D**), TOAs) in roots and coleoptile of 3-day-old seedlings of wheat (*Triticum aestivum* L. cv. ‘Ostka Strzelecka’). Control grains were germinated in double distilled water. Values are means (*n* = 3) + SD. Bars with the same letters (a–b, *a–c*) are not significantly (*p* < 0.05) different after the ANOVA test and Tukey’s post hoc corrections.

As the size of NPs increased (with the exception of 60 nm Ag NPs), there was also a slight decrease in the content of TAAs and TOAs in coleoptile (Figure 5C,D). Previously, we reported similar trends of changes in TIPM, TSC, TAA, and TOA in wheat seedling coleoptiles under increasing concentrations of (Bio)Ag NPs [48]. However, in roots, their concentration remained unchanged, regardless of the sizes of Ag NPs used (Figure 5). In endosperms, TIPMs concentration also remained almost unchanged.

### 2.3. The Effect of Ag NPs on Polar Metabolites in Wheat Seedlings

The effect of Ag NPs on changes in the concentrations of amino acids, organic acids, phosphate, and urea was almost not significant (Tables S1–S3). It means, that slight inhibition of early growth of wheat seedlings by citrate stabilized Ag NPs at 20 mg/L did not disturb nitrogen metabolism.

### 2.4. Changes in Metabolic Profiles of Polar Compounds under the Influence of Ag NPs

**Figure 6:**
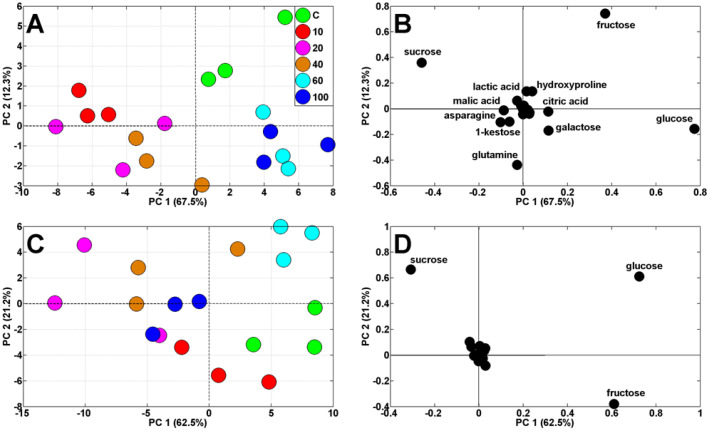
Principal components analysis (PCA) profiles of roots (**A**) and coleoptile (**C**) metabolites of wheat seedlings developing in dd water (control, **C**) and citrate-stabilized Ag NPs with sizes of 10, 20, 40, 60, and 100 nm (at a concentration of 20 mg/L each) and PCA loading plots of the polar metabolites (**B**,**D**).

Principal components analysis (PCA) of polar metabolites in wheat seedling tissues showed a shift in the distribution of results obtained for roots and coleoptiles (Figure 6).

The analytical results of control roots and those treated with larger Ag NPs (60 and 100 nm) were to the right of PC1, sharing 67.5% of the variability, whereas root samples developing in suspensions of smaller Ag NPs (10–40 nm) were to the left (Figure 6A). The samples of control coleoptiles and those treated with 60 nm Ag NPs were also to the right of PC1, sharing 62.5% of the variability (Figure 6C). However, the separation of endosperm samples was less evident, except for the location of control samples to the right-bottom corner of PCA plot (according to PC1 and PC2, sharing 89.2 and 7.64% of variability, respectively, Figure S2A, Supplementary Materials). The PCA of metabolites in roots and coleoptile of wheat seedlings showed that the distribution of samples was influenced mainly by changes in concentrations of sucrose, glucose, and fructose (Figure 6). Samples of endosperms were clearly separated by sucrose, glucose, and maltose (Figure S2B).

### 2.5. The Effect of Ag NPs on Soluble Carbohydrates Content and Profile in Wheat Seedlings

**Figure 7:**
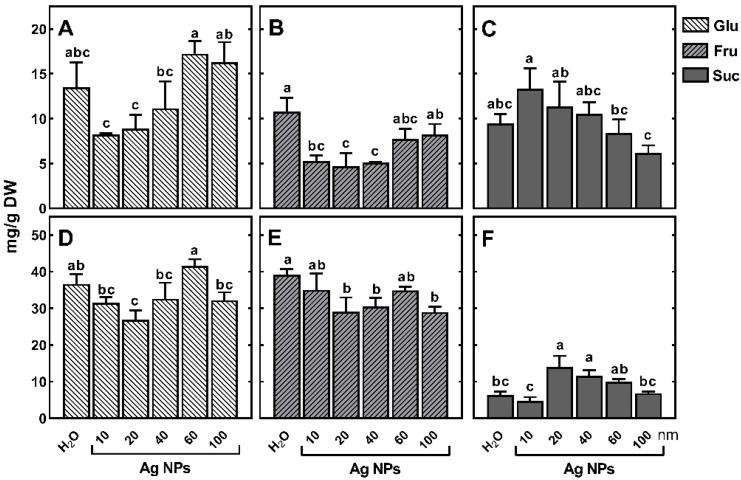
The effect of various sized Ag NPs citrate-stabilized nanoparticles in the range of 10–100 nm on the concentration of glucose (Glu), fructose (Fru), and sucrose (Suc) in roots (**A**–**C**) and coleoptile (**D**–**F**) of 3-day-old seedlings of wheat (*Triticum aestivum* L. cv. ‘Ostka Strzelecka’). Control grains were germinated in double distilled water. Values are means (*n* = 3) + SD. Bars with the same letters (a–c) are not significantly (*p* < 0.05) different after the ANOVA test and Tukey’s post hoc corrections.

Although the difference in the concentration of TSCs in roots of seedlings developing in Ag NPs suspensions relative to the control was not significant, considerable differences were shown in the content of monosaccharides and sucrose. In response to the small Ag NPs (10–40 nm), the concentrations of glucose and fructose were lower, while sucrose was higher than those in the control (Figure 7A–C).

In coleoptile, increased sucrose concentration in response to Ag NPs with the size of 20–60 nm coincided with a decrease in monosaccharides content (Figure 7D–F). The concentration of monosaccharides in coleoptile was ca 2-fold higher than that in roots (Figure 7A,B,D,E). In contrast, sucrose contents in roots and coleoptile of seedling treated with Ag NPs were similar (13.33 mg/g DW in roots in response to 10 nm Ag NPs, and 13.98 mg/g DW in coleoptile, in response to 20 nm Ag NPs, Figure 7C,D, Tables S1 and S2). These contents may be the result of inhibition of sucrose breakdown in slower-growing seedlings. However, the effect of Ag NPs (and/or released Ag^+^ ions) on the activity of enzymes participating in both sucrose synthesis and hydrolysis is not yet known.

Changes in the composition and concentration of soluble carbohydrates are closely related to the degradation of storage starch in the endosperm of germinating grain and further synthesis and transport of sucrose from scutellum into growing seedling [58]. The activity of α-amylase, a crucial enzyme for starch degradation during germination [63] can be both inhibited [64] or stimulated [65] by Ag NPs. In the case of α-amylase inhibition [63], it could be expected that the level of starch degradation products should be lower in the endosperm of Ag NPs-treated seedlings than in the control. However, such an effect was not found as the differences in the concentrations of total maltotriose, maltose, and glucose (in the range of 33.6–37.1 mg/g DW, Table S3) in the endosperm were not significant. On the other hand, the stimulation of α-amylase activity by Ag NPs should lead to an increase in the concentration of TSCs [65]. However, our study did not show this either (Figure 5B, Table S3). Similarly, the lack of effect of Ag NPs on α-amylase activity was last recently revealed in germinating alfalfa (Medicago sativa) seeds [66].

The highest concentration of sucrose in the root of seedlings treated with the low-sized Ag NPs was accompanied by the lowest concentration of sucrose in coleoptile (Figure 7C,F). This may be a result of Ag NPs-induced changes in sucrose distribution between endosperm, coleoptile, and roots under stress conditions. Moreover, sucrose accumulation in wheat seedlings both under (Bio)Ag NPs [48] and citrate-stabilized Ag NPs, in the present study, indicates the importance of sucrose metabolism in response to this type of stress. This has also been demonstrated in the response of pea seedlings to Ag^+^ ions [47]. All of these results suggest that sucrose metabolism plays a key role in the stress response to Ag NPs or Ag^+^ ions. Sucrose and glucose are signaling molecules that can regulate meristem activity, root growth, expression of various sugars-related genes, and auxin metabolism [67]. Therefore, maintaining sucrose/monosaccharides homeostasis under abiotic stress conditions can be essential for plant survival [68]. Additionally, sucrose plays an important role in the response of plants to osmotic stress.

Among other carbohydrates, galactose was found to be reduced in the roots of seedlings treated with 10 and 20 nm Ag NPs. (Table 1). This monosaccharide is derived from the hydrolysis of raffinose, present in dry wheat grains [69], and is a substrate for the synthesis of arabinogalactans in cell walls [70] as well as arabinogalactan proteins in wheat seedlings roots [71]. Additionally, the concentration of 1-kestose was higher than in control (5.5–6.0 and 4.6 mg/g DW, respectively), while myo-inositol and maltose remained unaffected, regardless of the NPs size (Table S1). This means that the major changes in carbohydrates in response to Ag NPs are related to sucrose metabolism.

## 3. Materials and Methods

### 3.1. Characteristics of Ag NPs

In the present study, the commercially available silver nanoparticles suspensions with sizes of 10, 20, 40, 60, and 100 nm (catalog numbers: 730785, 730793, 730807, 730815, and 730777, respectively, Sigma-Aldrich, Saint Louis, MO, USA) at a concentration of 20 mg/L each were used. The size and shape of the Ag NPs were confirmed using transmission electron microscopy (JEM-1400, JEOL, Tokyo, Japan) at 80 kV, whereas the elemental composition was analyzed using SEM/EDS (JSM-5310LV, JEOL, Japan, with EDS Noran System 7, Thermo-Scientific, Madison, Strzelce, WI, USA) at 25 kV.

### 3.2. The Effect of Ag NPs on Grains Germination and Development of Seedlings

Grains of wheat (*Triticum aestivum* L., cv. Ostka Strzelecka purchased from Hodowla Roślin Strzelce, Poland) were germinated on filter papers in Petri dishes (80 × 15 mm) containing 5 mL of double-distilled water (control) or Ag NPs suspensions at 20 °C in the dark. Each treatment was made in three replicates (each replicate is one Petri dish containing 20 grains). During 3 days of incubation, germinability (%) and the percentage of developing seedlings were calculated as in the previous study [48].

After 3 days, the length of the longest radicle and coleoptile was measured; the seedlings then were separated into coleoptile (including plumule), roots (including scutellum and mesocotyl), and endosperm (with seed coat), frozen in liquid nitrogen, stored in an ultra-refrigerator (at −80 °C) for 2 days, and then freeze-dried (shelf freeze-dryer, Alpha 1–2 LD, Martin Christ, Osterode am Harz, Germany) for 48 h. Dry tissues were pulverized (for 2 min at 22 Hz) in a mixed mill (MM 200, Retsch, Verder Scientific GmbH, Haan, Germany) for extraction of polar metabolites.

### 3.3. Generation of Reactive Oxygen Species and Cytotoxicity

The generation of reactive oxygen species and viable and non-viable cells in root tips of wheat seedlings developing in suspensions of Ag NPs were analyzed using confocal laser scanning microscopy (CLSM), as described previously [48].

### 3.4. Polar Metabolite Profiling

The extraction of polar metabolites from freeze-dried and pulverized tissues of wheat seedlings cv. Ostka Strzelecka was carried out as described previously [47,48]. Briefly, polar metabolites were extracted using the hot (70 °C) mixture of methanol: water, and then, the non-polar metabolites were removed by cold chloroform. After two-steps derivatization (with *O*-methoxamine hydrochloride and followed with the mixture of *N*-methyl-*N*-trimethylsilyl-trifluoroacetamide with pyridine), the TMS (*tri*-methylsilyl)-derivatives of polar metabolites were separated on a ZEBRON ZB-5MSi Guardian capillary column (Phenomenex, Torrance, CA, USA) in a gas chromatograph equipped with a flame ionization detector (FID) (GC-2010 Plus, Shimadzu, Kyoto, Japan). The polar metabolites were identified by the comparison of the retention time (RT) with the RT of original standards (Sigma-Aldrich, Saint-Louis, MO, USA). Moreover, for the confirmation of proper identification of metabolites, a GC-2010 coupled with a quadrupole mass spectrometry analyzer (GCMS-QP2010, Shimadzu, Kyoto, Japan) was used. Metabolites were identified by comparison of the RT, relative retention time (RRT), retention indices (RI) and mass spectra of original standards and from the NIST 05 library (National Institute of Standards and Technology, Gaithersburg, MD, USA). Data were collected and analyzed using GC-MS Solution and LabSolutions software (Shimadzu, Kyoto, Japan).

### 3.5. Statistical Analysis

The results (means of three independent replicates) were subjected to one-way ANOVA with a post hoc test (Tuckey, if overall *p* < 0.05) using Statistica software (version 12.0; StatSoft, Tulsa, OK, USA). Graphs were prepared using GraphPad Prism (version 3.0; GraphPad Software, San Diego, CA, USA). Principal component analysis (PCA) for multivariate statistics to compare the metabolic profiles of roots, coleoptiles, and endosperms of 3-day-old wheat seedlings in response to Ag NPs was performed using COVAIN program [72], a MATLAB toolbox including a graphical user interface (MATLAB version 2013a; Math Works, Natick, MA, USA).

## 4. Conclusions

Citrate-stabilized Ag NPs affect the central metabolism of wheat seedlings depending on nanoparticle size. The reason is probably that smaller Ag NPs are more easily absorbed by roots than larger ones. This causes growth inhibition of young wheat seedlings, increased generation of ROS and cells death in root tips, and changes in carbohydrate metabolism and/or transport. An increase in sucrose concentration in roots and coleoptile of 3-day-old seedlings, accompanied by a decrease in glucose and fructose, appears to be the first metabolic response to stress induced by Ag NPs. Other effects of Ag NPs on wheat seedlings remain to be investigated.

## Figures and Tables

**Table 1:** The concentration of total identified polar metabolites (TIPMs), total soluble carbohydrates (TSCs), total amino acids (TAAs), total organic acids (TOAs), and total remaining compounds (TRCs) in roots, coleoptile, and endosperm of 3-day-old control seedlings of wheat (*Triticum aestivum* L., cv. ‘Ostka Strzelecka’).

Metabolites	Roots	Coleoptile	Endosperm
	mg/g DW		
TIPMs, including:	107.70	129.69	56.08
TSCs, including:	45.17	94.58	51.30
fructose	10.78	39.11	0.17
glucose	13.52	36.65	3.75
galactose	5.00	4.37	0.47
*myo*-inositol	0.30	1.69	0.19
sucrose	9.46	6.36	10.65
maltose	1.54	1.12	31.83
maltotriose	-	-	1.54
1-kestose	4.57	5.28	2.69
TAAs, including:	35.61	17.16	3.00
alanine	0.80	1.09	0.13
asparagine	8.63	2.58	0.05
aspartic acid	0.15	0.31	0.01
GABA	0.46	1.80	0.09
glutamic acid	1.13	0.66	0.14
glutamine	11.27	2.98	0.21
glycine	0.52	0.79	0.11
hydroxyproline	4.86	1.84	0.31
isoleucine	1.14	0.19	0.13
leucine	0.86	0.08	0.15
lysine	0.42	1.30	0.16
methionine	0.04	0.04	0.01
phenylalanine	0.28	0.17	0.30
proline	1.70	1.02	0.52
serine	0.99	0.65	0.17
threonine	0.46	0.14	0.05
tryptophan	0.11	0.05	0.12
tyrosine	0.43	1.10	0.13
valine	1.36	0.39	0.22
TOAs, including:	19.70	9.09	1.11
citric acid	5.76	4.60	0.65
fumaric acid	0.12	0.36	0.01
malic acid	11.88	2.32	0.10
lactic acid	1.48	0.86	0.19
oxalic acid	0.42	0.77	0.15
propionic acid	0.04	0.18	0.01
TRC, including:	7.22	8.86	0.68
phosphoric acid	6.96	8.71	0.65
urea	0.26	0.16	0.03

## Data Availability

Not applicable.

## Supplementary Materials

The following supporting information can be downloaded at: https://www.mdpi.com/article/10.3390/ijms232113255/s1.
