# Supplementary material for: The Size-Dependent Effects of Silver Nanoparticles on Germination, Early Seedling Development and Polar Metabolite Profile of Wheat (Triticum aestivum L.)
Source: Int J Mol Sci. 2022 Oct 31;23(21):13255. doi: 10.3390/ijms232113255 (PMC9657336; doi:10.3390/ijms232113255)
Supplement: Supplementary file 1 [file ijms-23-13255-s001.zip › ijms-1929345-supplementary.pdf]

Supplementary materials

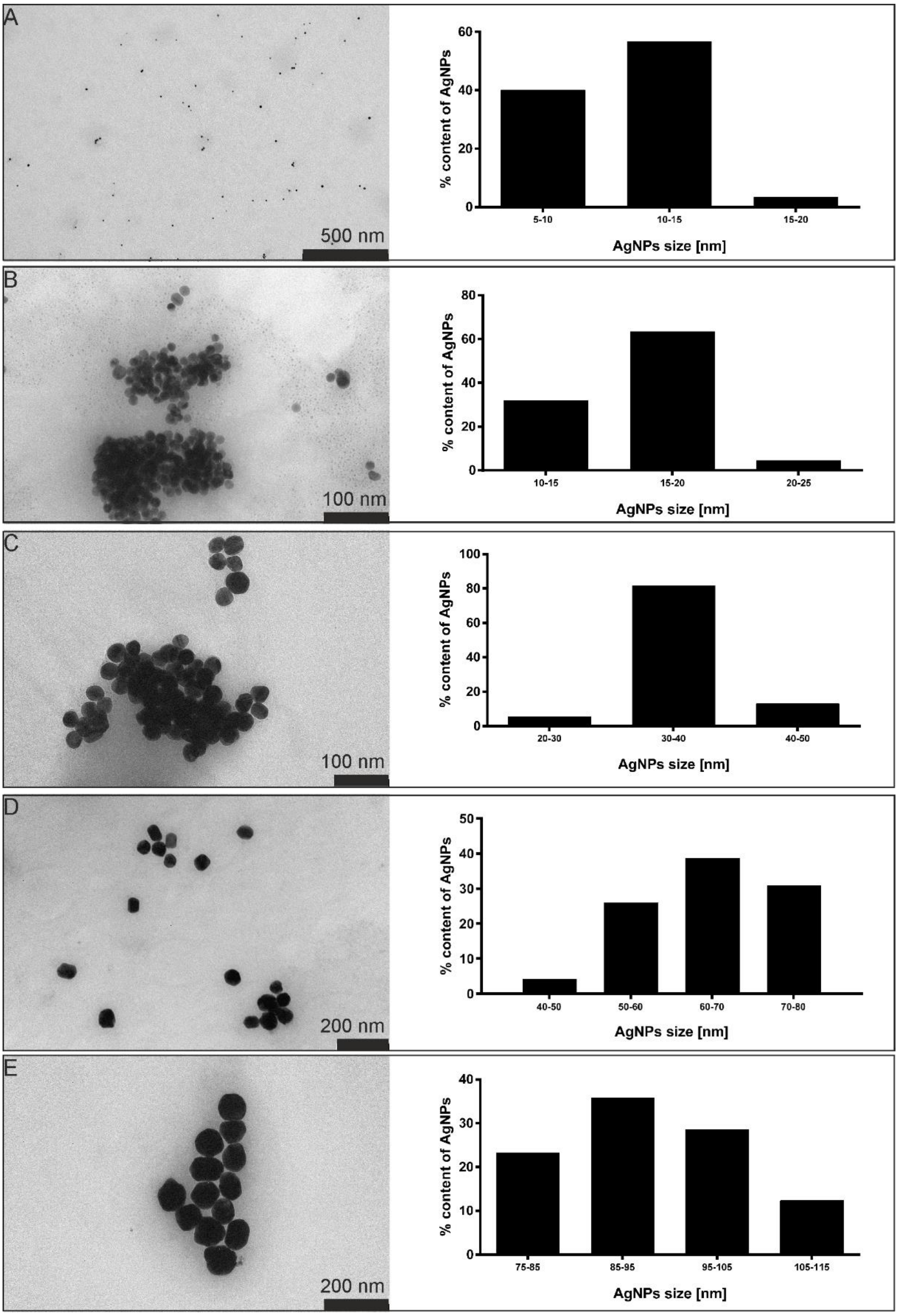

Figure S1. The size distribution of Ag NPs declared as 10, 20, 40, 60 and 100 nm (A, B, C, D, and E).

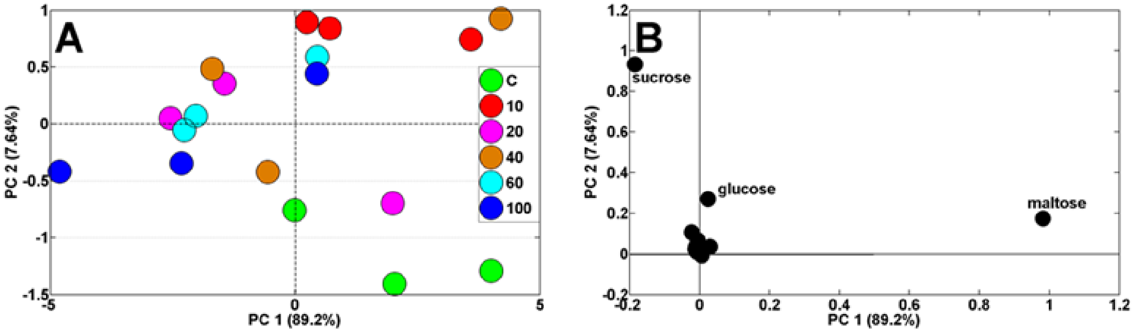

Figure S2. (A) PCA profiles of endosperm metabolites of wheat seedlings developing in double distilled water (control, *C*) and in the presence of citrate-stabilized Ag NPs with the size of 10, 20, 40, 60 and 100 nm (at the concentration of 20 mg/L each) and (B) PCA loading plots of the polar metabolites.

Table S1. The concentrations of polar metabolites, including total identified polar metabolites (TIPMs), total soluble carbohydrates (TSCs), total amino acids (TAAs), total organic acids (TOAs), and total remaining compounds (TRCs) in roots of 3-day-old seedling of wheat (*Triticum aestivum* L., cv. 'Ostka Strzelecka') developing in dd water (control) or in 20 mg/L Ag NPs with a different size (10, 20, 40, 60 and 100 nm). Means of 3 replicates. The same letters by the values indicate no statistically significant differences (P<0.05) based on ANOVA analysis and Tukey's post- hoc corrections.

**Roots**	**Control**	**Ag NPs, size, nm**				
	**(H_2O)**	10	20	40	60	100
**Metabolites**		mg/g DW				
TIPMs, including:	107.70^{a}	102.09^{a}	100.63^{a}	103.23^{a}	112.82^{a}	106.09^{a}
TSCs, including:	45.17^{a}	38.36^{a}	37.08^{a}	40.68^{a}	46.65^{a}	44.17^{a}
fructose	10.78^{a}	5.27^{bc}	4.67^{c}	5.13^{c}	7.73^{abc}	8.24^{ab}
glucose	13.52^{abc}	8.23^{c}	8.88^{c}	11.17^{bc}	17.26^{a}	16.31^{ab}
galactose	5.00^{abc}	3.80^{d}	4.57^{cd}	6.16^{a}	5.84^{ab}	5.73^{ab}
myo-inositol	0.30^{a}	0.26^{a}	0.24^{a}	0.27^{a}	0.29^{a}	0.26^{a}
sucrose	9.46^{abc}	13.33^{a}	11.37^{ab}	10.54^{abc}	8.39^{bc}	6.20^{c}
maltose	1.54^{a}	1.46^{a}	1.85^{a}	1.43^{a}	2.03^{a}	1.88^{a}
1-kestose	4.57^{b}	6.00^{a}	5.49^{ab}	5.98^{a}	5.11^{ab}	5.54^{a}
TAAs, including:	35.61^{a}	36.71^{a}	36.88^{a}	37.61^{a}	37.68^{a}	36.44^{a}
alanine	0.80^{a}	0.79^{a}	0.82^{a}	0.74^{a}	0.63^{a}	0.75^{a}
asparagine	8.63^{a}	10.16^{a}	9.97^{a}	8.65^{a}	8.94^{a}	8.83^{a}
aspartic acid	0.15^{b}	0.49^{ab}	0.37^{ab}	0.33^{ab}	0.56^{a}	0.35^{ab}
GABA	0.46^{a}	0.42^{a}	0.46^{a}	0.38^{a}	0.43^{a}	0.44^{a}
glutamic acid	1.13^{a}	1.08^{a}	1.09^{a}	0.95^{a}	0.89^{a}	0.96^{a}
glutamine	11.27^{a}	11.98^{a}	12.68^{a}	14.08^{a}	12.71^{a}	12.42^{a}
glycine	0.52^{a}	0.47^{ab}	0.46^{ab}	0.44^{b}	0.43^{b}	0.43^{b}
hydroxyproline	4.86^{a}	3.77^{b}	3.52^{b}	3.91^{ab}	3.77^{b}	4.24^{ab}
isoleucine	1.14^{a}	1.21^{a}	1.23^{a}	1.26^{a}	1.31^{a}	1.23^{a}
leucine	0.86^{a}	0.86^{a}	0.83^{a}	1.01^{a}	1.04^{a}	0.89^{a}
lysine	0.42^{a}	0.38^{a}	0.33^{a}	0.41^{a}	0.91^{a}	0.44^{a}
methionine	0.04^{a}	0.04^{a}	0.04^{a}	0.05^{a}	0.07^{a}	0.04^{a}
phenylalanine	0.28^{a}	0.21^{a}	0.23^{a}	0.23^{a}	0.26^{a}	0.24^{a}
proline	1.70^{ab}	1.59^{ab}	1.52^{b}	1.88^{ab}	2.07^{a}	1.66^{ab}
serine	0.99^{a}	0.98^{a}	0.97^{a}	0.94^{a}	1.01^{a}	0.93^{a}
threonine	0.46^{a}	0.50^{a}	0.48^{a}	0.43^{a}	0.44^{a}	0.46^{a}
tryptophan	0.11^{a}	0.09^{a}	0.09^{a}	0.06^{a}	0.10^{a}	0.10^{a}
tyrosine	0.43^{ab}	0.23^{b}	0.30^{ab}	0.32^{ab}	0.48^{ab}	0.50^{a}
valine	1.36^{b}	1.48^{ab}	1.50^{ab}	1.54^{ab}	1.62^{a}	1.52^{ab}
TOAs, including:	19.70^{a}	19.62^{a}	19.33^{a}	18.20^{a}	21.35^{a}	18.75^{a}
citric acid	5.76^{a}	5.00^{a}	5.11^{a}	5.19^{a}	6.73^{a}	6.30^{a}
fumaric acid	0.12^{a}	0.18^{a}	0.15^{a}	0.11^{a}	0.17^{a}	0.15^{a}
malic acid	11.88^{ab}	13.35^{a}	13.13^{ab}	11.80^{ab}	13.06^{ab}	11.37^{b}
lactic acid	1.48^{a}	0.54^{b}	0.47^{b}	0.67^{b}	0.55^{b}	0.45^{b}
oxalic acid	0.42^{a}	0.46^{a}	0.43^{a}	0.38^{a}	0.62^{a}	0.41^{a}
propionic acid	0.04^{a}	0.09^{a}	0.05^{a}	0.05^{a}	0.22^{a}	0.07^{a}
TRCs, including:	7.22^{a}	7.39^{a}	7.34^{a}	6.72^{a}	7.13^{a}	6.73^{a}
phosphoric acid	6.96^{a}	7.30^{a}	7.27^{a}	6.63^{a}	7.04^{a}	6.64^{a}
urea	0.26^{a}	0.09^{b}	0.08^{b}	0.09^{b}	0.09^{b}	0.09^{b}

Table S2. The concentrations of polar metabolites, including total identified polar metabolites (TIPMs), total soluble carbohydrates (TSCs), total amino acids (TAAs), total organic acids (TOAs), and total remaining compounds (TRCs) in coleoptile of 3-day-old seedling of wheat (*Triticum aestivum* L., cv. 'Ostka Strzelecka') developing in dd water (control) or in 20 mg/L Ag NPs with a different size (20, 40, 60 and 100 nm). Means of 3 replicates. The same letters by the values indicate no statistically significant differences (P<0.05) based on ANOVA analysis and Tukey's post- hoc corrections.

**Coleoptile**	**Control**	**Ag NPs, size, nm**				
	**(H_2O)**	10	20	40	60	100
**Metabolites**			mg/g DW			
TIPMs, including:	129.69^{ab}	119.41^{bc}	118.32^{bc}	118.36^{bc}	138.08^{a}	111.19^{c}
TSCs, including:	94.58^{ab}	93.52^{bc}	84.26^{bc}	88.05^{bc}	100.55^{a}	80.60^{c}
fructose	39.11^{a}	35.05^{ab}	29.04^{b}	30.45^{b}	34.85^{ab}	28.98^{b}
glucose	36.65^{ab}	31.49^{bc}	26.85^{c}	32.59^{bc}	41.56^{a}	32.21^{bc}
galactose	4.37^{a}	4.75^{a}	4.93^{a}	4.53^{a}	5.50^{a}	4.79^{a}
myo-inositol	1.69^{ab}	1.78^{ab}	1.69^{ab}	1.65^{ab}	1.97^{a}	1.63^{b}
sucrose	6.36^{bc}	4.69^{c}	13.98^{a}	11.64^{a}	9.93^{ab}	6.85^{bc}
maltose	1.12^{ab}	1.07^{b}	1.66^{a}	0.73^{b}	0.84^{b}	0.87^{b}
1-kestose	5.28^{a}	5.72^{a}	6.11^{a}	6.46^{a}	5.90^{a}	5.27^{a}
TAAs, including:	17.16^{a}	16.31^{a}	16.09^{a}	15.17^{a}	18.38^{a}	14.22^{a}
alanine	1.09^{a}	0.90^{a}	0.83^{a}	0.97^{a}	1.01^{a}	0.87^{a}
asparagine	2.58^{ab}	2.34^{ab}	2.22^{b}	2.35^{ab}	3.19^{a}	2.43^{ab}
aspartic acid	0.31^{ab}	0.27^{b}	0.28^{ab}	0.33^{ab}	0.39^{a}	0.35^{ab}
GABA	1.80^{ab}	1.67^{ab}	1.62^{ab}	1.51^{b}	1.94^{a}	1.61^{ab}
glutamic acid	0.66^{a}	0.75^{a}	0.70^{a}	0.66^{a}	0.58^{a}	0.51^{a}
glutamine	2.98^{ab}	3.22^{a}	3.73^{a}	2.83^{ab}	3.22^{a}	1.12^{b}
glycine	0.79^{ab}	0.80^{a}	0.80^{a}	0.68^{ab}	0.80^{a}	0.65^{b}
hydroxyproline	1.84^{a}	1.84^{a}	1.57^{a}	1.49^{a}	1.93^{a}	1.71^{a}
isoleucine	0.19^{ab}	0.20^{ab}	0.19^{ab}	0.18^{b}	0.23^{a}	0.19^{b}
leucine	0.08^{a}	0.08^{a}	0.10^{a}	0.08^{a}	0.11^{a}	0.08^{a}
lysine	1.30^{a}	1.19^{a}	0.86^{a}	0.78^{a}	1.05^{a}	1.73^{a}
methionine	0.04^{a}	0.04^{a}	0.04^{a}	0.03^{a}	0.03^{a}	0.03^{a}
phenylalanine	0.17^{a}	0.16^{a}	0.16^{a}	0.14^{a}	0.19^{a}	0.12^{a}
proline	1.02^{abc}	0.82^{c}	1.03^{abc}	1.53^{ab}	1.58^{a}	0.98^{bc}
serine	0.65^{ab}	0.62^{ab}	0.63^{ab}	0.53^{b}	0.70^{a}	0.57^{ab}
threonine	0.14^{b}	0.15^{ab}	0.16^{ab}	0.14^{b}	0.19^{a}	0.13^{b}
tryptophan	0.05^{ab}	0.04^{ab}	0.06^{a}	0.05^{ab}	0.05^{ab}	0.02^{b}
tyrosine	1.10^{a}	0.85^{ab}	0.72^{ab}	0.52^{b}	0.71^{ab}	0.76^{ab}
valine	0.39^{ab}	0.37^{b}	0.38^{ab}	0.39^{ab}	0.49^{a}	0.36^{b}
TOAs, including:	9.09^{ab}	9.42^{ab}	9.13^{ab}	7.76^{b}	10.21^{a}	8.55^{ab}
citric acid	4.60^{a}	5.03^{a}	4.96^{a}	3.93^{a}	5.43^{a}	4.58^{a}
fumaric acid	0.36^{ab}	0.40^{a}	0.38^{b}	0.27^{b}	0.40^{b}	0.31^{ab}
malic acid	2.32^{ab}	2.70^{a}	2.46^{ab}	1.87^{c}	2.18^{bc}	2.18^{bc}
lactic acid	0.86^{a}	0.57^{a}	0.64^{a}	0.70^{a}	1.07^{a}	0.57^{a}
oxalic acid	0.77^{a}	0.53^{a}	0.53^{a}	0.81^{a}	0.91^{a}	0.74^{a}
propionic acid	0.18^{a}	0.19^{a}	0.17^{a}	0.17^{a}	0.22^{a}	0.18^{a}
TRCs, including:	8.86^{a}	9.13^{a}	8.85^{a}	7.37^{b}	8.94^{a}	7.82^{ab}
phosphoric acid	8.71^{ab}	9.01^{a}	8.74^{a}	7.24^{b}	8.80^{a}	7.72^{ab}
urea	0.16^{a}	0.12^{a}	0.10^{a}	0.13^{a}	0.14^{a}	0.10^{a}

Table S3. The concentrations of polar metabolites, including total identified polar metabolites (TIPMs), total soluble carbohydrates (TSCs), total amino acids (TAAs), total organic acids (TOAs), and total remaining compounds (TRCs) in endosperm of 3-day-old seedling of wheat (*Triticum aestivum* L., cv. 'Ostka Strzelecka') developing in dd water (control) or in 20 mg/L Ag NPs with a different size (20, 40, 60 and 100 nm). Means of 3 replicates. The same letters by the values indicate no statistically significant differences (P<0.05) based on ANOVA analysis and Tukey's post- hoc corrections.

**Endosperm**	**Control (H_2O)**	**Ag NPs size, nm**				
		10	20	40	60	100
**Metabolite**	mg/g DW					
TIPMs, including:	56.08[a]	59.64[a]	56.02[a]	58.03[a]	56.40[a]	55.27[a]
TSCs, including:	51.30[a]	54.09[a]	50.69[a]	52.49[a]	50.76[a]	49.68[a]
fructose	0.17[b]	0.26[a]	0.20[ab]	0.20[ab]	0.21[ab]	0.22[ab]
glucose	3.75[b]	4.77[a]	3.97[ab]	4.33[ab]	3.77[a]	4.21[ab]
galactose	0.47[ab]	0.50[ab]	0.51[ab]	0.38[b]	0.62[a]	0.52[ab]
myo-inositol	0.19[a]	0.21[a]	0.21[a]	0.19[a]	0.21[a]	0.21[a]
sucrose	10.65[b]	12.48[a]	12.14[ab]	12.23[ab]	12.59[a]	12.34[ab]
maltose	31.83[a]	31.65[a]	29.39[a]	30.74[a]	28.86[a]	27.83[a]
maltotriose	1.54[a]	1.54[a]	1.40[a]	1.57[a]	1.52[a]	1.55[a]
1-kestose	2.69[a]	2.70[a]	2.88[a]	2.84[a]	2.98[a]	2.80[a]
TAAs, including:	3.00[b]	3.60[a]	3.46[ab]	3.62[a]	3.74[a]	3.73[a]
alanine	0.13[bc]	0.15[abc]	0.15[a]	0.14[abc]	0.12[c]	0.13[c]
asparagine	0.05[a]	0.06[a]	0.06[a]	0.07[a]	0.07[a]	0.06[a]
aspartic acid	0.01[a]	0.01[a]	0.01[a]	0.01[a]	0.01[a]	0.01[a]
GABA	0.09[a]	0.12[a]	0.13[a]	0.08[a]	0.09[a]	0.10[a]
glutamic acid	0.14[c]	0.14[c]	0.15[bc]	0.17[abc]	0.20[a]	0.19[ab]
glutamine	0.21[b]	0.26[ab]	0.26[ab]	0.29[ab]	0.32[a]	0.31[a]
glycine	0.11[b]	0.14[a]	0.13[ab]	0.11[ab]	0.12[ab]	0.12[ab]
hydroxyproline	0.31[b]	0.39[ab]	0.37[ab]	0.42[a]	0.39[ab]	0.40[ab]
isoleucine	0.13[b]	0.15[a]	0.15[a]	0.15[a]	0.15[a]	0.15[a]
leucine	0.15[a]	0.17[a]	0.15[a]	0.16[a]	0.19[a]	0.18[a]
lysine	0.16[a]	0.19[a]	0.19[a]	0.20[a]	0.22[a]	0.23[a]
methionine	0.01[a]	0.02[a]	0.01[a]	0.01[a]	0.02[a]	0.02[a]
phenylalanine	0.30[b]	0.34[ab]	0.32[ab]	0.33[ab]	0.34[ab]	0.34[a]
proline	0.52[b]	0.63[a]	0.58[ab]	0.62[a]	0.62[a]	0.60[ab]
serine	0.17[b]	0.20[a]	0.19[ab]	0.20[a]	0.20[a]	0.21[a]
threonine	0.05[a]	0.06[a]	0.06[a]	0.06[a]	0.05[a]	0.05[a]
tryptophan	0.12[a]	0.12[a]	0.12[a]	0.13[a]	0.13[a]	0.15[a]
tyrosine	0.13[c]	0.19[bc]	0.18[bc]	0.21[ab]	0.26[a]	0.25[a]
valine	0.22[b]	0.24[a]	0.23[ab]	0.24[a]	0.23[ab]	0.23[ab]
TOAs, including:	1.11[a]	1.06[a]	1.09[a]	1.11[a]	1.11[a]	1.05[a]
citric acid	0.65[a]	0.60[a]	0.62[a]	0.68[a]	0.71[a]	0.64[a]
fumaric acid	0.01[a]	0.01[a]	0.01[a]	0.01[a]	0.01[a]	0.01[a]
malic acid	0.10[b]	0.12[ab]	0.13[ab]	0.11[ab]	0.14[a]	0.13[ab]
lactic acid	0.19[a]	0.16[abc]	0.17[ab]	0.16[bcd]	0.13[d]	0.14[cd]
oxalic acid	0.15[ab]	0.15[a]	0.15[ab]	0.14[ab]	0.11[b]	0.12[b]
propionic acid	0.01[a]	0.01[a]	0.01[a]	0.01[a]	0.01[a]	0.01[a]
TRCs, including:	0.68[b]	0.88[a]	0.78[ab]	0.81[ab]	0.80[ab]	0.80[ab]
phosphoric acid	0.65[b]	0.86[a]	0.75[ab]	0.78[ab]	0.77[ab]	0.78[ab]
urea	0.03[a]	0.03[a]	0.03[a]	0.03[a]	0.03[a]	0.03[a]
